# A survey on the effectiveness of WhatsApp for teaching doctors preparing for a licensing exam

**DOI:** 10.1371/journal.pone.0231148

**Published:** 2020-04-02

**Authors:** Bankole K. Oyewole, Victor J. Animasahun, Helena J. Chapman

**Affiliations:** 1 Faculty of Clinical Sciences, Obafemi Awolowo College of Health Sciences, Olabisi Onabanjo University, Sagamu, Ogun State, Nigeria; 2 Science and Technology Policy Fellow, American Association for the Advancement of Science, Washington, D.C., United States of America; Leiden University Medical Center, NETHERLANDS

## Abstract

**Introduction:**

The use of WhatsApp for health professional education is not novel and is described increasingly in literature as an affordable, familiar, and convenient tool for collaboration. Social media technologies for health practitioner education allow the use of text and audio-visual aids, peer-to-peer based learning, and problem-based learning. This study presents a survey on the effectiveness of WhatsApp in doctors’ preparation for a medical licensing exam.

**Methods:**

A cross-sectional study was conducted among one hundred and ninety-four participants of a WhatsApp group preparing for the PLAB exam over a three-month period. A twenty-item questionnaire designed on Google Form was completed by consenting participants on the publication of exam results.

**Results:**

Of the one hundred and ninety-four participants, one hundred and fourteen met the eligibility criteria, 57.9 percent were male and 42.1 percent were female, aged between twenty-four and forty-three years of age ($\bar{\text{x}}=30.6\pm 4.6$ years). A total of 88.6 percent of participants passed the exam in contrast to the global average pass rate of 69 percent, while the average score among participants was 131.5 compared to a global average score of 128. Passing the exam was significantly associated with combining the WhatsApp group with the online question bank Plabable (p = 0.001). While the mean age of those who passed the PLAB exam was lower than those who did not pass the exam, the number of years post-graduation had no significant association with passing the exam. A total of 93.8 percent stated that moderators were knowledgeable, 83.3 percent reported that the platform increased their motivation to learn, 72.8 percent felt that the session were organised and easy-to-follow, and 97.4 percent of participants reported they would recommend the PLAB network.

**Conclusion:**

WhatsApp can be an effective tool for health professional education, using a pre-defined curriculum coupled with organizational structure. This study reported both subjective and objective measures of effectiveness and demonstrated that the use of multiple e-learning resources can lead to improved learning outcomes.

## Introduction

The frequency and utility of real-time social media tools (e.g., smartphone apps) in classroom and community settings has been a fundamental addition to medical education.[1] With an increase in the use of innovative technology for learning, these tools have been instrumental in providing medical students with the timely access to clinical information, such as laboratory values or pharmaceutical side effects.[2]Medical students have incorporated social media platforms (e.g., YouTube, Facebook, WhatsApp) in their e-learning, using online question banks and study aids to supplement their academic learning.[3] The evaluation of these social media tools and platforms in medical students’ preparation of formal standardized examinations (e.g., national medical board certifications) may highlight an overlooked benefit in the learning process.

WhatsApp is a free social media platform based on mobile instant messaging (MIM) that facilitates the creation of groups of participants and sharing of text messages, multimedia files, and other documents. The use of WhatsApp for health professional education is not novel and has been described in preparation of a psychiatry membership exam, academic courses in histology and ophthalmology, and primary health education activities. Social media technologies allow the use of text and audio-visual aids, peer-to-peer based learning, and problem-based learning. They provide a platform that stimulates interaction, collaboration and participation through the sharing ideas and experience, this has led participants to report positive outcomes in the use of WhatsApp as a learning aid.[4–7]

The Professional and Linguistics Assessments Board examination (PLAB), organised by the General Medical Council (GMC), is the test that international medical graduates (IMGs) complete to be considered for registration to the United Kingdom’s medical register and subsequently be granted a general license to practise. The examination consists of two parts. The first part is a multiple-choice question (MCQ) consisting of 180 questions, while the second part is an objectively-structured clinical examination (OSCE) made up of eighteen scenarios.[8] In 2018, 7,559 candidates completed the PLAB 1 examination in over twenty centres across the world.[9] This represented an increase of over two thousand candidates writing the exam, when compared to the previous year. Notably, unlike the United States Medical Licensing Exam (USMLE), the GMC does not offer exam preparation courses to medical graduates.

In September 2016, a WhatsApp group named “PLAB Network” was created as a forum, where doctors preparing for their PLAB 1 examination could discuss, exchange ideas, and share relevant practise materials, experience, and knowledge. Participants were added to the group based on recommendations from previous or current members and an eligible passing score on the International English Language Testing System (IELTS). This study aimed to assess the effectiveness of WhatsApp social media technology in preparing physicians to obtain a passing score on the medical licencing exam. The secondary study objective was to identify factors that were associated with physicians obtaining a passing exam score.

## Methodology

### Study design

In this cross-sectional study, a twenty-item survey was administered to one hundred and ninety-four participants WhatsApp group participants during a two-week period (April 27 to May 11, 2019). Inclusion criteria are those WhatsApp participants who completed the PLAB 1 on March 14, 2019. Exclusion criteria being participation in the group for less than the two weeks.

### Description of the WhatsApp platform

This gratuitous WhatsApp platform serves as a biannual forum, with an average of two hundred and forty participants and ten moderators, where questions are discussed and answered using approved references. Forum sessions are dedicated to specific clinical subjects (e.g., electrocardiogram interpretation, neurology, medical ethics) and other general topics (e.g., academic and clinical learning gaps). Due to limitations of this social media platform, only two hundred and fifty-six participants can join one WhatsApp group.

Sessions started approximately three months prior to the PLAB 1 examination date, initially lasting one hour daily and then increased to three hours daily during weekdays, between the hours of 20:00 and 23:00. Using a compiled question bank, practice questions were discussed using a problem-solving approach, with participation of a facilitator and two moderators. Moderators were unpaid volunteers who have been previous participants of the group that have shown a keen interest in teaching and had a passing score of the PLAB 1 examination. This platform allowed the sharing of references from GMC approved websites, images that helped to explain anatomical or clinical scenarios were also shared and discussed, website links to relevant guidelines and information like that of the National Institute for Health and Care Excellence (NICE) were attached to relevant explanations, PowerPoint presentations developed by moderators on topics like interpretation of electrocardiograms and ethics were provided to participants and participants were also sign posted to relevant book chapters and paragraphs that contained detailed information on scenarios. These facilitated collective discussion. After-hours posts were reviewed, and queries were answered the following weekday.

### Dataset description and questionnaire design

The questionnaire was designed on the virtual Google Form, consisting of twenty questions with fourteen mandatory questions necessary for submitting the form. There were three sections: demography, exam preparation, and perception of group effectiveness. The demography section contained five questions which covered participant biodata and level of training, the section on exam preparation contained five questions assessing participants score, study materials and duration of preparation while the section on perception of group effectiveness contained ten questions assessing respondent’s perception of group organization, motivation to learn, level of intrusiveness and recommendations.

Authors BKO and VJA independently conducted comprehensive literature reviews to identify germane questions that assessed effectiveness of social media in medical education and agreed upon a final list of items included in the survey questions which was then pretested and finalized, these ensured the validity and reliability of the questionnaire. No personal information data (e.g., names, email) were collected.

### Data analysis

Data on group participants was downloaded from Google Forms into Microsoft Excel and subsequently coded and transferred into the IBM Statistical Package for Social Sciences (SPSS) version 25. Data on global statistics on average pass marks and rate was extracted from the GMC website. Descriptive statistics were calculated in form of frequencies, percentages, means, and standard deviation. Inferential statistics were calculated, and the level of significance was set at 5 percent. Independent sample t-test was used to calculate the mean difference of variables. Chi-square test and Fisher’s exact test were used to determine associations between categorical variables.

### Ethical considerations

The authors performed the study independently, outside of their current institutional affiliations. However, ethical standards were maintained following the applicable principles of the World Medical Association Declaration of Helsinki.[10] Informed consent was obtained by study participants, and data were anonymised. Data collection complied with WhatsApp–s terms and conditions.

## Results

### General characteristics of the respondents

Out of the one hundred and ninety-four participants of the PLAB network, a total of one hundred and fourteen respondents met the eligibility criteria and completed the online survey after informed consent was obtained. Of the respondents, 57.9 percent were male and 42.1 percent were females, ranging between twenty-four and forty-three years of age ($\bar{\text{x}}=30.6\pm 4.6$ years). A total of 61.4 percent was within the first five years post-graduation, 28.9 percent were within six to ten years post-graduation, and 9.6 percent had graduated from medical school over ten years. Respondents were described at different stages of their medical career, including house officer (5.3 percent), non-training role (66.7 percent), training post (26.3 percent), and consultant (1.8 percent).

### PLAB result of participants in the PLAB network

Of the total 180 points set by the GMC, respondents scored between 84 and 154 points ($\bar{\text{x}}=131.5\pm 12.7$ points). As the GMC passing score was 120, 88.6 percent participants passed the PLAB exam with 120 points or above, however 64.9 percent scored the average score of 128 or above.

### Factors associated with passing the PLAB exam

Of those respondents, the mean age of those who passed the PLAB exam was lower than those who did not pass the exam (30.3±4.4 vs 33.2±5.8 years) (t = 2.16; p = 0.03). Other factors had no significant association with passing the PLAB exam, including mean duration of preparation in months (t = -0.41; p = 0.68), average study hours per day (t = -0.71; p = 0.48), and number of years post-graduation (X^2^ = 3.314; p = 0.19).

Passing the PLAB exam was significantly associated with using the following as the main study resource: Plabable (an online question bank) (p = 0.009) and combining PLAB network with Plabable (p = 0.001). Passing the PLAB exam was not significantly associated with using the following as main study resource: PLAB network (p = 0.23); 1700 questions (p = 0.77); pass medicine (p = 1.00); others (e.g. textbooks, internet resources, telegram groups, lecture notes (p = 1.00); combining PLAB network with 1700 questions (p = 0.42); combining PLAB network with pass medicine (an online question bank) (p = 0.33); and combining PLAB network with other resources (p = 0.19) (Table 1).

**Table 1:** Association between main study resource(s) and passing the PLAB exam.

Variable	Total n = 114(%)	Pass n = 101(%)	Fail n = 13(%)	Test Statistic
**PLAB Network**				
Yes	101(83.3)	86(85.1)	9(69.2)	X^2^ = 2.10
				p = 0.23^+^
No	13(16.7)	15(14.9)	4(30.8)	
**1700 Questions**				
Yes	67(58.8)	60(59.4)	7(53.8)	X^2^ = 0.15
				p = 0.77^+^
No	47(41.2)	41(40.6)	6(46.2)	
**Plabable**				
Yes	86(75.4)	80(79.2)	6(46.2)	X^2^ = 6.80
				p = 0.009*
No	28(24.6)	21(20.8)	7(53.8)	
**Pass medicine**				
Yes	2(1.8)	2(2.0)	0(0.0)	X^2^ = 0.26
				p = 1.00^+^
No	112(98.2)	99(98.0)	13(100.0)	
**Others**				
Yes	14(12.3)	13(12.9)	1(7.7)	X^2^ = 0.29
				p = 1.00^+^
No	100(87.7)	88(87.1)	12(92.3)	
**PLAB Network + 1700 Questions**				
Both	63(55.3)	58(57.4)	5(38.5)	X^2^ = 1.79
				p = 0.42^+^
Either	36(31.6)	30(29.7)	6(46.2)	
Neither	15(13.2)	13(12.9)	2(15.4)	
**PLAB Network + Plabable**				
Both	72(63.2)	67(66.3)	5(38.5)	X^2^ = 13.27
				p = 0.001*
Either	37(32.5)	32(31.7)	5(38.5)	
Neither	5(4.4)	2(2.0)	3(23.1)	
**PLAB Network + Pass Medicine**				
Both	2(1.8)	2(2.0)	0(0)	X^2^ = 2.28
				p = 0.33^+^
Either	93(81.6)	84(83.2)	9(69.2)	
Neither	19(16.7)	15(14.9)	4(30.8)	
**PLAB Network + Others**				
Both	10(8.8)	10(9.9)	0(0)	X^2^ = 2.39
				p = 0.19^+^
Either	79(78.1)	79(78.2)	10(76.9)	
Neither	12(13.2)	12(11.9)		

* p<0.05 was considered statistically significant

^+^ Fisher’s exact test when cell count was less than 5

### Participants’ perceptions about the PLAB network learning platform

Of the respondents, 83.3 percent reported that the platform increased their motivation to learn, 80.7 percent believed that the platform encouraged collaborative learning and participation, and 93.8 percent stated that moderators were knowledgeable. Over two-thirds (68.4 percent) mentioned that they preferred learning through this WhatsApp platform, and 72.8 percent felt that the sessions were organised and easy-to-follow. However, about half (50.9 percent) felt the level of irrelevant messages were either low or very low, 27.2 percent felt it was average, and 22 percent) felt that it was high or very high. (Table 2). A total of 97.4 percent of participants said that they would recommend the PLAB network to any colleague who planned to prepare for an upcoming PLAB exam.

**Table 2:** Participants’ feedback about the PLAB network learning platform.

Variable	Strongly Disagree n(%)	Disagree n(%)	Neither agree not disagree n(%)	Agree n(%)	Strongly Agree n(%)
It increased my motivation to learn	1(0.9)	2(1.8)	16(14.0)	27(23.7)	68(59.6)
It encouraged collaborative learning and participation	1(0.9)	3(2.6)	18(15.8)	39(34.2)	53(46.5)
The moderators were knowledgeable	1(0.9)	1(0.9)	5(4.4)	39(34.2)	68(59.6)
I prefer learning through this method	3(2.6)	4(3.5)	29(25.4)	34(29.8)	44(38.6)
Sessions were organised and easy to follow	4(3.5)	3(2.6)	24(21.1)	36(31.6)	47(41.2)
	Very Low n(%)	Low n(%)	Average n(%)	High n(%)	Very High n(%)
What was the level of irrelevant messages during the discussions	34(39.8)	24(21.1)	31(27.2)	19(27.2)	6(5.3)

## Discussion

WhatsApp is believed to be one of the most preferred mobile messaging applications for interaction, communication, and learning.[11] Our study uniquely assessed both qualitative and quantitative measures of effectiveness in the use of WhatsApp in healthcare professional education. The gender distribution (57.9 percent male, 42.1 percent female) was similar to that (58.4 percent male, 41.6 percent female) of IMGs writing the United States Medical Licensing Examination (USMLE) exam and that that (54 percent male, 46 percent female) of all doctors registered with the GMC.[12,13] The mean age (30.6 years) of respondents was also similar that (30.8 years) of IMGs taking the USMLE exam.[12]

The mean score of participants of the WhatsApp teaching in our study was higher than the general GMC average, and the majority (88.6 percent) passed the exam. There is evidence that these interactive, web chat-based social media platforms can improve learning experience and enhance knowledge acquisition, which was exemplified by neurology case-based discussions and problem-based learning among medical students in Nigeria and the United Kingdom.[14,15] However, one study among medical students in India reported no significant difference in knowledge among respondents when WhatsApp learning was compared with didactic lectures.[16] Notably, in this study, combining the WhatsApp learning with an independent online resource (Plabable) was most beneficial for passing the PLAB exam, rather than using the PLAB network WhatsApp learning platform as the only study resource. This was similar to findings of a quasi-experimental study design among selected secondary school teenagers, where a significant increase in post-intervention scores was observed with teenagers who had supplementary learning on WhatsApp coupled with traditional classroom learning, versus only received classroom lessons.[11] Hence, this suggests that the WhatsApp teaching coupled with secondary learning strategies have the potential to enhance an individual’s success on standard exams like PLAB exam.

Respondents’ perceived level of intrusive messages during WhatsApp teaching sessions was higher than previous studies.[14,15] This might reflect the peer-led nature of our study, as opposed to other studies where professional hierarchy might help ensure decorum. Although posting of irrelevant messages is a known disadvantage of WhatsApp learning platforms,[7] we believe that by setting clear ground rules and giving warnings or sanctions may maximize focus and productivity.

With increased interest in the use of WhatsApp in medical education, more than seventy-five percent of articles on this topic have been published in the last three years.[17] This reflects both the popularity of the use of convenient mobile platforms like WhatsApp for medical education among healthcare professionals and also the need for data on their effectiveness among researchers.[11,17]

Since its creation in 2016, the PLAB Network has remained popular WhatsApp platform among doctors writing the PLAB exam. Based on participants’ recommendations, the group has reached maximum capacity for its three-monthly biannual sessions over the last three years. An average of more than seven hundred and fifty doctors have participated in the group over this period. Participants reported positive feedback, agreeing that it fosters collaborative learning as a hub for sharing relevant study resources. The group–s effectiveness was demonstrated via a positive perception from participants and observation of higher pass rates.

This study has several limitations. First, nonresponse bias may have resulted, since participants who achieved passing or failing scores may have varying perspectives of the effectivity of the WhatsApp platform. Second, the variability of their participation, whether for partial or complete duration, may have affected their perception of the effectiveness. Third, due to the homogenous study population, cultural differences could not be explored in this study, limiting the generalization of study findings to other population groups.

Finally, teaching methods via each WhatsApp group may vary and influence the effectiveness as a teaching tool. However, a pre-defined curriculum coupled with organizational structure can harness social media technologies for medical education.

## Conclusion

The study examined the use of social media technology among medical graduates as they prepared for a medical licensing examination. Studies among other population groups have reported positive or non-significant outcomes, which highlights the need for further research in social media applications related to medical education.

Social media technologies like WhatsApp are a convenient way for healthcare practitioners to collaboratively prepare and acquire essential information and resources to pass licensing examinations. This study reported both positive quantitative and qualitative measures of effectiveness related to the WhatsApp tool. Study findings demonstrated that the use of multiple e-learning resources, including social media technology, may lead to improvement in learning outcomes.

## Supporting information

S1 Data (DOCX)

## References

[1] Animasahun VJ, Chapman HJ, Oyewole BK. Social media to guide ‘One Health’ initiatives. *Clin Teach* 2019;16: 1–3. 10.1111/tct.13045 31240806

[2] Davies BS, Rafique J, Vincent TR, Fairclough J, Packer MH, Vincent R, et al Mobile medical education (MoMEd)-how mobile information resources contribute to learning for undergraduate clinical students-a mixed methods study. *BMC Med Educ* 2012;12 (1):1. 22240206 10.1186/1472-6920-12-1 PMC3317860

[3] Wynter L, Burgess A, Kalman E, Heron J, Bleasel J. Medical students: what educational resources are they using? *BMC Med Educ* 2019;19: 36 10.1186/s12909-019-1462-9 30683084 PMC6347772

[4] Willemse JJ. Undergraduate nurses’ reflections on Whatsapp use in improving primary health care education. *Curationis* 2015;38 (2):1–7. 10.4102/curationis.v38i2.1512 PMC6091559 26304053

[5] Loo JL, Koh EB, Pang NT, Nor Hadi NM. Use of WhatsApp in assisting psychiatry learning. *Med Educ* 2016;50 (11):1165. 10.1111/medu.13195 27762032

[6] Dar QA, Ahmad F, Ramzan M, Khan SH, Ramzan K, Ahmed W, et al Use of social media tool “Whatsapp” in medical education. *Annals* 2017;23 (1).

[7] Maske SS, Kamble PH, Kataria SK, Raichandani L, Dhankar R. Feasibility, effectiveness, and students’ attitude toward using WhatsApp in histology teaching and learning. *J Edu Health Promot* 2018;7: 158. 10.4103/jehp.jehp_30_18 PMC6332667 30693295

[8] General Medical Council. A guide to the PLAB test. https://www.gmc-uk.org/registration-and-licensing/join-the-register/plab/a-guide-to-the-plab-test. 2019 [cited 19 May 2019].

[9] General Medical Council. Recent pass rates for PLAB 1 and PLAB 2. https://www.gmc-uk.org/registration-and-licensing/join-the-register/plab/recent-pass-rates-for-plab-1-and-plab-2. 2019 [cited 19 May 2019].

[10] World Medical Association. Declaration of Helsinki. Ethical principles for medical research involving human subjects. *Bull World Health Organ* 2001;79 (4):373–4. 11357217 PMC2566407

[11] Cetinkaya L. The impact of Whatsapp use on success in education process. *International Review of Research in Open and Distributed Learning* 2017;18 (7).

[12] Van Zanten M, Boulet JR, McKinley DW, DeChamplain A, Jobe AC. Assessing the communication and interpersonal skills of graduates of international medical schools as part of the United States medical licensing exam (USMLE) step 2 clinical skills (CS) exam. *Acad Med* 2007;82 (10 Suppl):S65–8. 10.1097/ACM.0b013e318141f40a 17895694

[13] General Medical Council. Data explorer. https://data.gmc-uk.org/gmcdata/home/#/reports/The%20Register/Stats/report. 2019 [cited 16 July 2019].

[14] Adebayo PB, Taiwo FT. Chat group helps improve the effect of neurophobia. *World Neurology* 2017;32 (5):13.

[15] Raiman L, Antbring R, Mahmood A. WhatsApp messenger as a tool to supplement medical education for medical students on clinical attachment. *BMC Med Educ* 2017;17 (1):7 10.1186/s12909-017-0855-x 28061777 PMC5219809

[16] Gon S, Rawekar A. Effectivity of e-learning through Whatsapp as a teaching learning tool. *MVP Journal of Medical Sciences* 2017;4 (1):19–25.

[17] Coleman E, O’Connor E. The role of WhatsApp^®^ in medical education; a scoping review and instructional design model. *BMC Med Educ* 2019;19 (1):279 10.1186/s12909-019-1706-8 31345202 PMC6659203

