# Supplementary material for: A survey on the effectiveness of WhatsApp for teaching doctors preparing for a licensing exam
Source: PLoS One. 2020 Apr 2;15(4):e0231148. doi: 10.1371/journal.pone.0231148 (PMC7117696; doi:10.1371/journal.pone.0231148)
Supplement: S1 Data — (DOCX) [file pone.0231148.s001.docx]

PLAB NETWORK Feedback

1. Sex (Male, Female) *
2. Age*
3. Year of qualification*
4. Level of training (House officer, non-training role, training post/resident, consultant, other) *
5. Speciality of interest
6. PLAB1 Score*
7. What was your main study resource? * (PLAB Network, 1700 questions, Plabable, pass medicine, academy, others)
8. How many months did you prepare for?
9. Average study hours per day
10. Number of previous attempts
11. The PLAB Network helped me prepare for the exam* ( 1 strongly disagree 2 disagree 3 neither agree nor disagree 4 agree 5 strongly agree)
12. It increased motivation to learn* ( 1 strongly disagree 2 disagree 3 neither agree nor disagree 4 agree 5 strongly agree)
13. It encouraged collaborative learning and participation* ( 1 strongly disagree 2 disagree 3 neither agree nor disagree 4 agree 5 strongly agree)
14. The moderators were knowledgeable* ( 1 strongly disagree 2 disagree 3 neither agree nor disagree 4 agree 5 strongly agree)
15. I prefer learning through this method* ( 1 strongly disagree 2 disagree 3 neither agree nor disagree 4 agree 5 strongly agree)
16. The sessions were organised and easy to follow* ( 1 strongly disagree 2 disagree 3 neither agree nor disagree 4 agree 5 strongly agree)
17. Level of intrusive messages ( 1 Very low 2 low 3 moderate 4 high 5 Very high)
18. I would recommend the PLAB Network (Yes, No, Maybe)
19. Why are you writing the exam?
20. Anything else?

*-Mandatory question on google forms needed to complete the form

Google forms allows for the inclusion of a short explanation for each question and grouping them into sections
